# CircHIPK3 prevents chondrocyte apoptosis and cartilage degradation by sponging miR‐30a‐3p and promoting PON2

**DOI:** 10.1111/cpr.13285

**Published:** 2022-06-18

**Authors:** Jie Shang, Huizi Li, Biao Wu, Ning Jiang, Bin Wang, Dawei Wang, Junlong Zhong, Yufeng Chen, Xianghe Xu, Huading Lu

**Affiliations:** ^1^ Department of Orthopaedics The Fifth Affiliated Hospital of Sun Yat‐Sen University Zhuhai China; ^2^ Guangdong Provincial Key Laboratory of Biomedical Imaging The Fifth Affiliated Hospital of Sun Yat‐Sen University Zhuhai China; ^3^ Department of General Surgery The Second Affiliated Hospital of Nanchang University Nanchang China; ^4^ Department of Orthopedics The Affiliated Hospital of Qingdao University Qingdao China; ^5^ Department of Orthopedics The First Affiliated Hospital of Nanchang University Nanchang China

## Abstract

Osteoarthritis (OA) is a common joint disease featured by the deterioration of articular cartilage and chondrocyte death. Emerging evidence has indicated that circular RNAs (circRNAs) play an essential role in OA progress. Here, we found that the expression of circHIPK3 was significantly decreased in human and mouse OA cartilage. Knocking down circHIPK3 increased apoptosis and intracellular ROS level in HC‐a chondrocytes. We performed proteomic studies and identified that circHIPK3 regulated chondrocyte apoptosis through the mitochondrial pathway. Results of JC‐1 staining and western blot further confirmed that mitochondrial outer membrane permeabilization was promoted in HC‐a chondrocytes transfected by circHIPK3 siRNA. In terms of mechanism, we showed that PON2 functioned as a potential target of circHIPK3 to regulate chondrocyte apoptosis. Moreover, we revealed that circHIPK3 interacted with miR‐30a‐3p to regulate PON2 expression in chondrocytes. Taken together, our findings suggested that circHIPK3 regulated chondrocyte apoptosis by mitochondrial pathway, and targeting the circHIPK3/miR‐30a‐3p/PON2 axis might be a potential strategy for OA treatment.

## Graphical abstract

The current study revealed the important role of circHIPK3 in regulating chondrocyte apoptosis and maintaining extracellular matrix (ECM) homeostasis. Mechanistically, circHIIPK3 might serve as a sponge of miR‐30a‐3p to regulate PON2 expression. The downregulation of circHIIPK3 resulted in the increased expression of miR‐30a‐3p and decreased expression of PON2, thus leading to mitochondrial pathway apoptosis and ECM destruction.

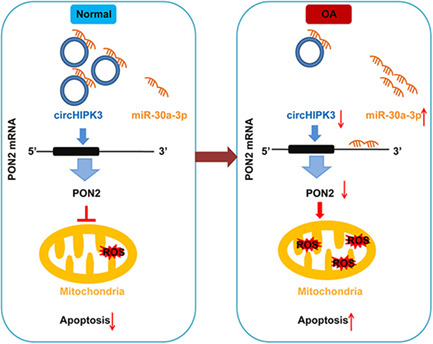

## 1 INTRODUCTION

Osteoarthritis (OA) is a common form of chronic arthritis prevalent in aged people, characterized by the destruction of articular cartilage.^1^ Articular cartilage is composed of chondrocytes and extracellular matrix (ECM). Chondrocytes, the unique cell population of cartilage, maintain the ECM equilibrium by secreting matrix proteins such as type II collagen (COL2A1) and aggrecan, as well as matrix‐degrading enzymes like matrix metalloproteinase 13 (MMP13).^2^, ^3^ Chondrocytes play an essential role in cartilage destruction and the pathology of OA. During OA progression, deterioration of chondrocytes causes the disruption of the metabolic balance of ECM and leads to loss of matrix proteins.^4^

Apoptosis is a process of programmed cellular death,^5^ which plays a critical role in the maintenance of tissue homeostasis.^6^ Chondrocyte apoptosis is essential for the development and growth of growth plates.^7^ However, in articular cartilage, chondrocyte apoptosis has been demonstrated to be positively correlated with cartilage destruction.^8^ Reactive oxygen species (ROS) induced oxidative damage is an important factor leading to cell apoptosis. The increment of ROS released by impaired chondrocytes^9^ promotes the expressions of MMPs like MMP13 and causes the destruction of cartilage.^10^ Excessive ROS generation is closely related to mitochondrial dysfunction. In apoptotic chondrocytes, decreased mitochondrial membrane potential leads to mitochondrial outer membrane permeabilization (MOMP) and the release of cytochrome c (Cyt‐c), which activates Caspase 3, the key enzyme of apoptosis. MOMP is known to be a key event of apoptosis, which can be triggered by the imbalance of anti‐apoptotic (e.g., BCL‐2) and pro‐apoptotic (e.g., BAX) members.^11^, ^12^, ^13^, ^14^ All of these suggest that mitochondria play a pivotal role in chondrocyte apoptosis.

circRNA is a class of endogenous RNA featuring for covalently closed loop structure without 5′ or 3′ polarities.^15^ Recently, circRNA, with various biological functions, has been involved in OA progress by regulating ECM formation, inflammation, apoptosis, and so on.^16^, ^17^ Philipp et al. analyzed the expressions of circRNAs in different MSC‐derived tissues, finding that among the top 10% differentially expressed circRNAs, circHIPK3 was significantly highly expressed in chondrocytes.^18^ CircHIPK3 (CircBase_ID:has_circRNA_000284) is generated from exon2 of the HIPK3 gene. Recent studies have revealed the important functional role of circHIPK3 in multiple diseases, such as colorectal cancer,^19^ diabetes,^20^ liver cancer,^21^ and pulmonary fibrosis.^22^ However, the role of circHIPK3 in OA is still enigmatic.

MicroRNAs are characterized as a class of small non‐coding RNA molecules 19–25 nucleotides in length. The main role of miRNAs is to regulate gene expression through translation repression or degradation of the mRNA by binding the 3′‐untranslated region (3′ UTR).^23^ It has been suggested that multiple miRNAs were involved in the pathogenesis of OA.^24^, ^25^, ^26^ Zhong et al. found that miRNA‐335‐5p could alleviate inflammation in human OA chondrocytes by activating autophagy.^25^ Li et al. reported that miRNA‐103 regulated chondrocyte apoptosis by downregulation of SPHK1.^26^

Paraoxonase 2 (PON2) is a member of the paraoxonases family, which contains PON1‐3.^27^ PON2 is extensively expressed in multiple tissues and involved in the processes of various diseases, such as Alzheimer's disease,^28^ atherosclerosis,^29^, ^30^ and cancers.^31^, ^32^ Accumulating evidence revealed the functional role of PON2 in antioxidative,^33^ anti‐apoptosis,^34^ and anti‐inflammation.^35^ It has been demonstrated that PON2 could specifically reduce superoxide release from the inner mitochondrial membrane.^36^ In PON2 deficient mice, enhanced mitochondrial oxidative stress was observed accompanied by significantly reduced electron transport chain complex I and III activities and decreased ATP levels.^30^ In oral squamous cell carcinomas (OSCC), knocking down PON2 significantly increased the irradiation‐induced apoptosis rates while upregulation of PON2 could protect OSCC against apoptosis.^34^ In this study, we revealed that circHIPK3 expression was decreased in OA chondrocytes and regulated apoptosis through mitochondria‐mediated pathway via miR‐30a‐3p/PON2 axis in chondrocytes. Furthermore, overexpression of circHIPK3 decreased the cartilage degeneration and suppressed chondrocyte apoptosis in OA mouse model.

## 2 MATERIALS AND METHODS

### 2.1 Human cartilage samples and experimental OA in mice

Human cartilage samples were obtained from seven OA patients and six non‐OA patients undergoing arthroplasty. All patients were provided with written informed consent before the operative procedure. The human cartilage sample collection and related experiments were approved by the Ethical Committee of the Fifth Affiliated Hospital of Sun Yat‐sen University and conducted according to the Helsinki Declaration guidelines. 12‐week‐old male C57BL/6 mice were purchased from Guangdong Medical Laboratory Animal Center to construct destabilization of the medial meniscus (DMM) model as previously described.^37^ To analyze the effect of circHIPK3 in vivo, lentivirus overexpressing circHIPK3 (CircBase ID:mmu_circ_0001052) (Purchased from Genepharma, Shanghai, China) were injected intra‐articularly (5 × 10^6^TU in a total volume of 10 μl) at 1/3/5 weeks after DMM surgery. Mice were sacrificed 8 weeks after DMM surgery to harvest knee joints and perform histological analysis. All mouse‐related experiments were approved by the Institutional Animal Care and Use Committee (IACUC) of Fifth Affiliated Hospital of Sun Yat‐sen University.

### 2.2 Chondrocyte culture and cell transfection

Human primary chondrocytes (HC‐a, #4650) were purchased from ScienCell Research Laboratories. HC‐a chondrocytes were cultured in Chondrocyte Medium (ScienCell, #4651) under standard cell culture conditions of 5% CO_2_ and 95% humidity. HC‐a chondrocytes within the fifth passage were used for experiments. With regard to cell transfection, HC‐a chondrocytes were transfected with circHIPK3 siRNA (Guangzhou Geneseed Biotech, Guangzhou, China), miR‐30a‐3p mimic, and inhibitor (Guangzhou RiboBio, Guangzhou, China) and PON2 siRNA (GenePharma, Shanghai, China) using Lipofectamine 3000 (Invitrogen) following the protocol of the manufacturer. The sequences of circHIPK3 and PON2 siRNA are shown in Table S1.

### 2.3 Flow cytometry analysis

HC‐a chondrocytes were washed with pre‐chilled PBS and collected 48 h after transfection to perform flow cytometry analysis. For apoptosis analysis, HC‐a chondrocytes were stained for Annexin V and propidium iodide (PI) using FITC Annexin V Apoptosis Detection Kit I (BD Pharmingen™) according to the manufacturer's protocol. To detect intracellular ROS levels, HC‐a chondrocytes were stained with fluorescent probe H_2_DCFDA (Invitrogen) according to the manufacturer's protocol. Flow cytometry was performed on CytoFLEX LX Flow Cytometer (Beckman Coulter), and data were analyzed by FlowJo software (Treestar, Ashland, OR).

### 2.4 Mitochondrial membrane potential (ΔΨm) assay

At 48 h after transfection, HC‐a chondrocytes were washed with PBS and incubated in fresh culture medium. ΔΨm of HC‐a chondrocytes were detected by a mitochondrial membrane potential assay kit with JC‐1 (Beyotime Biotechnology, China). HC‐a chondrocytes were incubated with JC‐1 in an incubator at 37°C for 20 min. After incubation, HC‐a chondrocytes were washed by pre‐chilled buffer twice and resuspended with fresh culture medium. The cells were examined with confocal laser microscopy (Zeiss, Oberkochen, Germany).

### 2.5 Real‐time quantitative PCR

Total RNA from articular cartilage and HC‐a chondrocytes were extracted by HP Total RNA Kit and EZNA Total RNA kit I (Omega Bio‐Tek, USA) respectively, according to the protocols of the manufacturer. After extraction of total RNA, cDNA was synthesized using random primers and RevertAid First Strand cDNA Synthesis kit (Thermo Scientific, USA). RT‐qPCR was performed by Forget‐Me‐NotTM Evergreen qPCR Master Mix (Biotium, CA) with a CFX96 TouchTM Real‐Time PCR Detection System (Bio RAD, USA). Reactions were conducted in a 10 μl reaction mixture and were incubated for 2 min at 95°C, followed by 40 cycles of a three‐step amplification procedure composed of denaturation for 5 s at 95°C, annealing for 10 s at 60°C, and extension for 10 s at 72°C. For quantitative results, the relative expression level of each mRNA, and circRNA was calculated using the 2^−ΔΔCt^ method. Student's *t*‐tests were applied and *p* < 0.05 was considered to be significant. Primers for RT‐qPCR reaction are listed in Table S1.

### 2.6 Western blot analysis

For isolation of cell proteins, HC‐a chondrocytes were washed with cold PBS twice and treated with RIPA buffer (Solarbio Biotech, Beijing, China). Cell lysates containing an equal amount of proteins were separated by electrophoresis and then transferred onto PVDF membranes (Immunoblot, Bio‐Rad). The membranes were blocked in TBST containing 5% non‐fat milk (Difco™ Skim Milk, BD) for 1 h at room temperature. After blocking, the membranes were incubated with primary antibodies against COL2A1 (Santa Cruz, USA), MMP13 (Santa Cruz, USA), PON2 (Abcam, USA), BCL2 (CST, USA), BAX (CST, USA), Cytochrome C (Abcam, USA), GAPDH (CST, USA) at 4 °C overnight, and then incubated with horseradish peroxidase(HRP)‐conjugated secondary antibody (Santa Cruz, USA). The antibody binding was detected using an ECL Western Blotting Substrate (Solarbio Science & Technology) and visualized using a Molecular Imager ChemiDoc XRS System (Bio‐Rad).

### 2.7 Binding site prediction and dual‐luciferase reporter assay

To predict the relationship among circHIPK3, miR‐30a‐3p and PON2, miRanda (www.microrna.org), and circBank (http://www.circbank.cn/) were employed. Then wild type or mutated sequences of circHIPK3 or PON2 were inserted into psiCHECK2 vectors (Geneseed Biotech, Guangzhou, China). 293T cells were co‐transfected with recombinant vectors and miR‐30a‐3p mimic or mimic NC for 48 h. After incubation, the luciferase signals were analyzed with a dual‐luciferase reporter assay system (Promega) according to the protocol. The relative luciferase activities were measured by normalizing the firefly luciferase to Renilla luciferase activities.

### 2.8 Histology, TUNEL, and immunohistochemistry

At the 8 weeks after treated DMM surgery, the knee joints were isolated and fixed with 4% paraformaldehyde in PBS at 4°C overnight. After decalcification in 10% EDTA for 3 weeks at 4°C, the tissues were embedded in paraffin. The paraffin blocks were cut at 5 μm thickness and deparaffinized in xylene, hydrated with graded ethanol and stained with safranin O‐fast green. The destruction of cartilage was scored according to the OARSI system (grade 0–6).^38^ Apoptosis was detected using the Tunel kit (Beyotime Biotechnology, China) according to the manufacturer's instructions. PON2 expression was detected in the mouse cartilage section by immunohistochemistry.

### 2.9 Statistical analysis

Data were presented as the mean ± SD. Statistical analysis was performed by Student's t‐test or one‐way analysis of variance (ANOVA). The data shown were representative results from three or more independent experiments. *p* < 0.05 was considered statistically significant.

## 3 RESULTS

### 3.1 CircHIPK3 was downregulated in OA chondrocytes and cartilage

First, we identified the existence of circHIPK3 in HC‐a chondrocytes. The result of Sanger sequencing verified the head‐to‐tail splicing in the RT‐PCR product of circHIPK3 (Figure S1a). Next, to further confirm the sequence and junction of circHIPK3, we performed RT‐PCR with the convergent and divergent primer of circHIPK3 and GAPDH using cDNA and genomic DNA (gDNA) from HC‐a chondrocytes. The electrophoresis of RT‐PCR revealed that circHIPK3 was only amplified by divergent primer in cDNA but not by convergent and divergent primer in gDNA (Figure S1b). Moreover, we performed the RNase R digestion assay to confirm the circular form of circHIPK3. RT‐qPCR showed that circHIPK3 was resistant to RNase R, while the liner RNA of HIPK3 was significantly decreased (Figure S1c). These results confirmed the existence of circHIPK3 in HC‐a chondrocytes.

**FIGURE 1:**
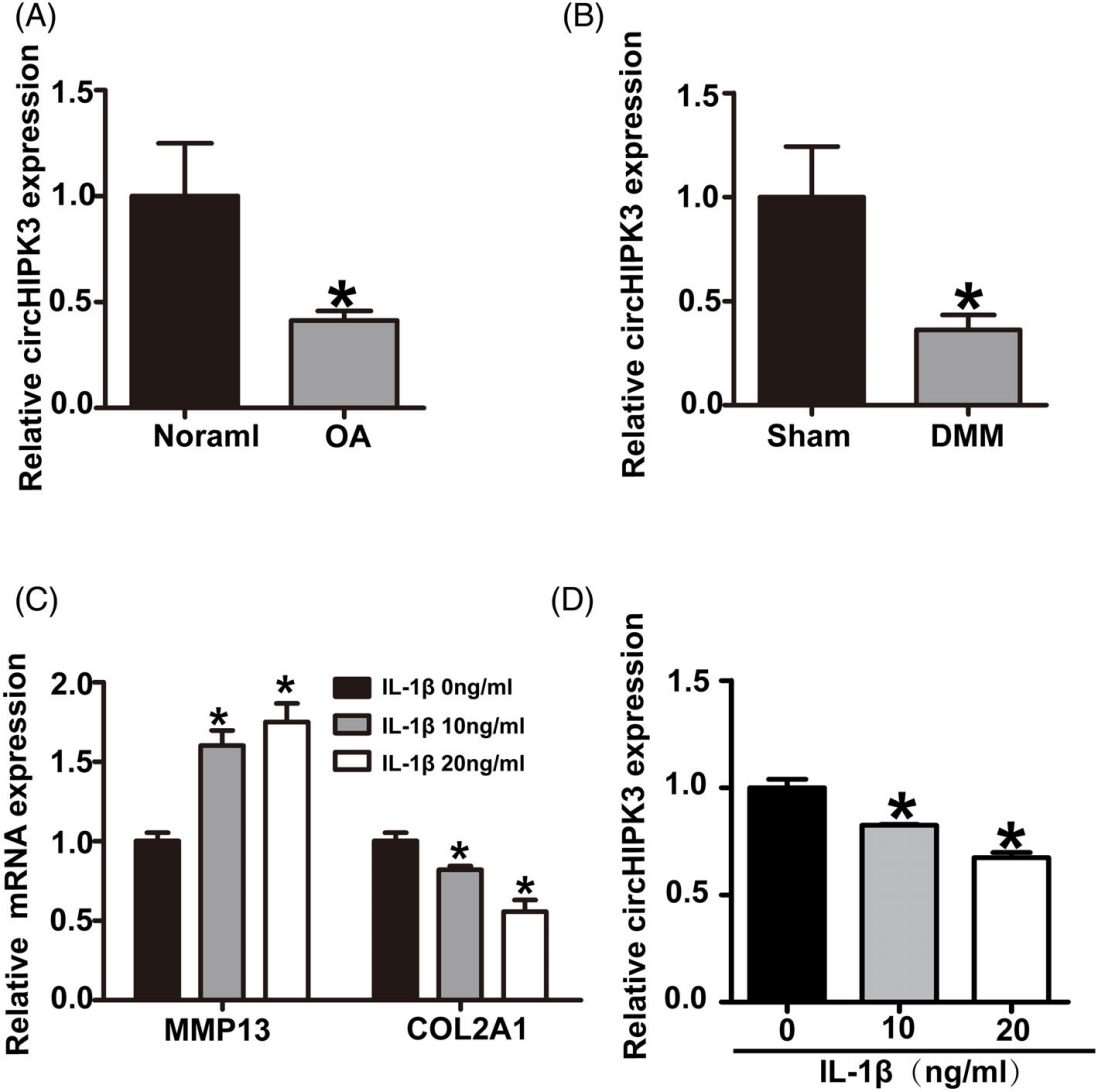
circHIPK3 was downregulated in OA. (A) RT‐qPCR analysis showed relative circHIPK3 expression in healthy and OA human cartilage tissue (7 OA tissues vs. 6 healthy tissues). (B) RT‐qPCR analysis showed relative circHIPK3 expression in sham and DMM mouse cartilage tissue (*n* = 4). (C, D) RT‐qPCR analysis showed expressions of COL2A1, MMP13 and circHIPK3 in HC‐a chondrocytes treated by IL‐1β for 48 h. *p < 0.05

To explore the role of circHIPK3 during OA progress, we first detected the expression of circHIPK3 in OA and normal cartilage. The results showed that circHIPK3 was downregulated in OA cartilage (Figure 1A). Decreased circHIPK3 was also confirmed in experimental mouse cartilage. CircHIPK3 (CircBase ID:has_circ_000284) was highly conserved with mmu_circ_0001052.^20^ Consistently, the expression of mmu_circ_0001052 was also decreased in DMM groups compared to that in sham groups (Figure 1B). We also characterized the expression of circHIPK3 in HC‐a chondrocytes stimulated with IL‐1β. After treating with IL‐1β for 48 h, COL2A1 was dose‐dependently decreased while MMP13 was significantly increased (Figure 1C). Notably, the expression of circHIPK3 was also dose‐dependently decreased after the stimulation of IL‐1β (Figure 1D).

### 3.2 Downregulated expression of circHIPK3 promoted cartilage matrix destruction

**FIGURE 2:**
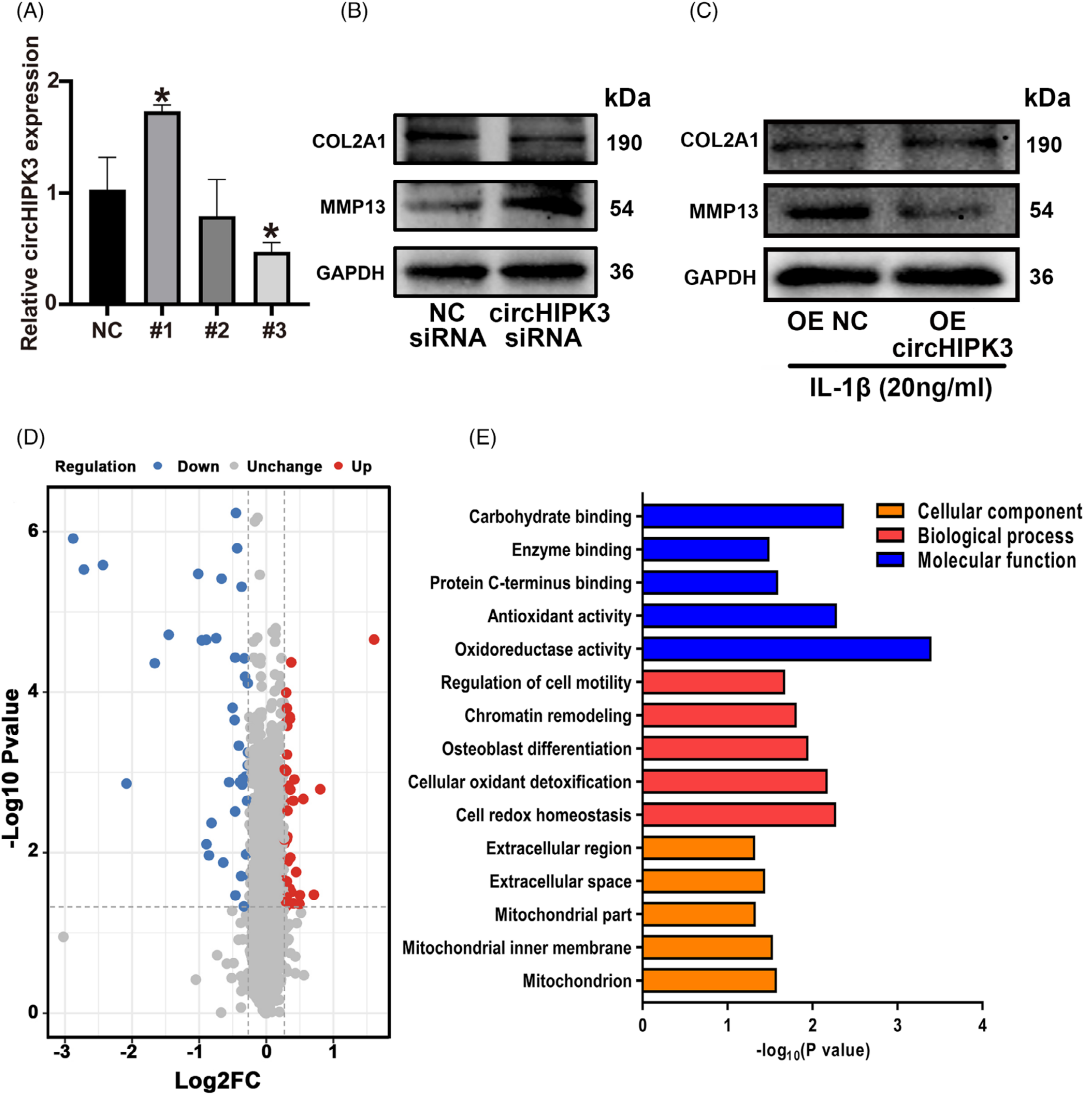
circHIPK3 regulated ECM degradation and circHIPK3‐related differentially expressed proteins were identified. (A) RT‐qPCR analysis showed relative circHIPK3 expression in HC‐a chondrocytes transfected by circHIPK3 siRNA #1,#2 and #3. (B) Western blot analysis showed COL2A1, MMP13 protein expressions in HC‐a chondrocytes transfected with circHIPK3 siRNA. (C) Western blot analysis showed COL2A1, MMP13 protein expressions in IL‐1β treated HC‐a chondrocytes transfected with circHIPK3 overexpression plasmid. (D) Volcano plots were constructed to show fold change values and *p* values. The vertical dotted lines show 1.2‐fold upregulation and downregulation between circHIPK3 siRNA and NC siRNA group, and the horizontal dotted line represents *p*‐value. (E) Differentially expressed proteins were enriched GO terms covering biological processes, cellular components and molecular functions. *p < 0.05

To investigate the functional role of circHIPK3 in cartilage, we performed knockdown experiments using siRNA specific to circHIPK3 (#1, #2, and #3). Results of RT‐qPCR displayed that circHIPK3 siRNA#3 has the best knockdown efficacy and thus was selected for further functional study (termed as circHIPK3 siRNA thereafter) (Figure 2A). Since OA was characterized by the imbalance between catabolism and anabolism of ECM, we analyzed the expressions of COL2A1 and MMP13 in HC‐a chondrocytes after transfection of circHIPK3 siRNA. Western blot revealed that downregulation of circHIPK3 inhibited COL2A1 expression but promoted MMP13 expression in HC‐a chondrocytes (Figure 2B). Besides, overexpression of circHIPK3 with plasmid led to the upregulation of COL2A1 and the downregulation of MMP13 in IL‐1β treated HC‐a chondrocytes (Figure 2C). These results indicated that the downregulation of circHIPK3 in chondrocytes contributed to the destruction of the ECM while overexpressing circHIPK3 protected against ECM degradation.

### 3.3 Screening and analyzing circHIPK3‐regulated proteins

To determine the mechanism of circHIPK3 involved in OA, we performed the proteomic analysis in HC‐a chondrocytes transfected with circHIPK3 siRNA. A total of 5731 proteins were identified, among which 80 proteins were dysregulated after knocking down circHIPK3 in HC‐a chondrocytes (fold change >1.2 and *p*‐value < 0.05). Of these 80 proteins, 40 proteins were downregulated and the other 40 proteins were upregulated (Figure 2D and Table S2).

To categorize the altered circHIPK3‐related proteins, we conducted Gene Ontology analysis. In the biological process and molecular function ontology, terms about the oxidative stress response, such as cell redox homeostasis, cellular oxidant detoxification, oxidoreductase activity, and antioxidant activity were highly enriched. Besides, in the category of cellular component ontology, some items related to mitochondria, such as mitochondria, mitochondrial inner membrane, and mitochondrial part were enriched (Figure 2E and Table S3). The oxidative stress response is known to be related to the destruction of ECM, and mitochondrial dysfunction is an important factor of oxidative stress in chondrocytes.^10^, ^39^ Thus, we speculated whether circHIPK3 regulated oxidative stress by affecting mitochondrial function.

### 3.4 CircHIPK3 regulated mitochondrial ROS production and apoptosis of HC‐a chondrocyte

**FIGURE 3:**
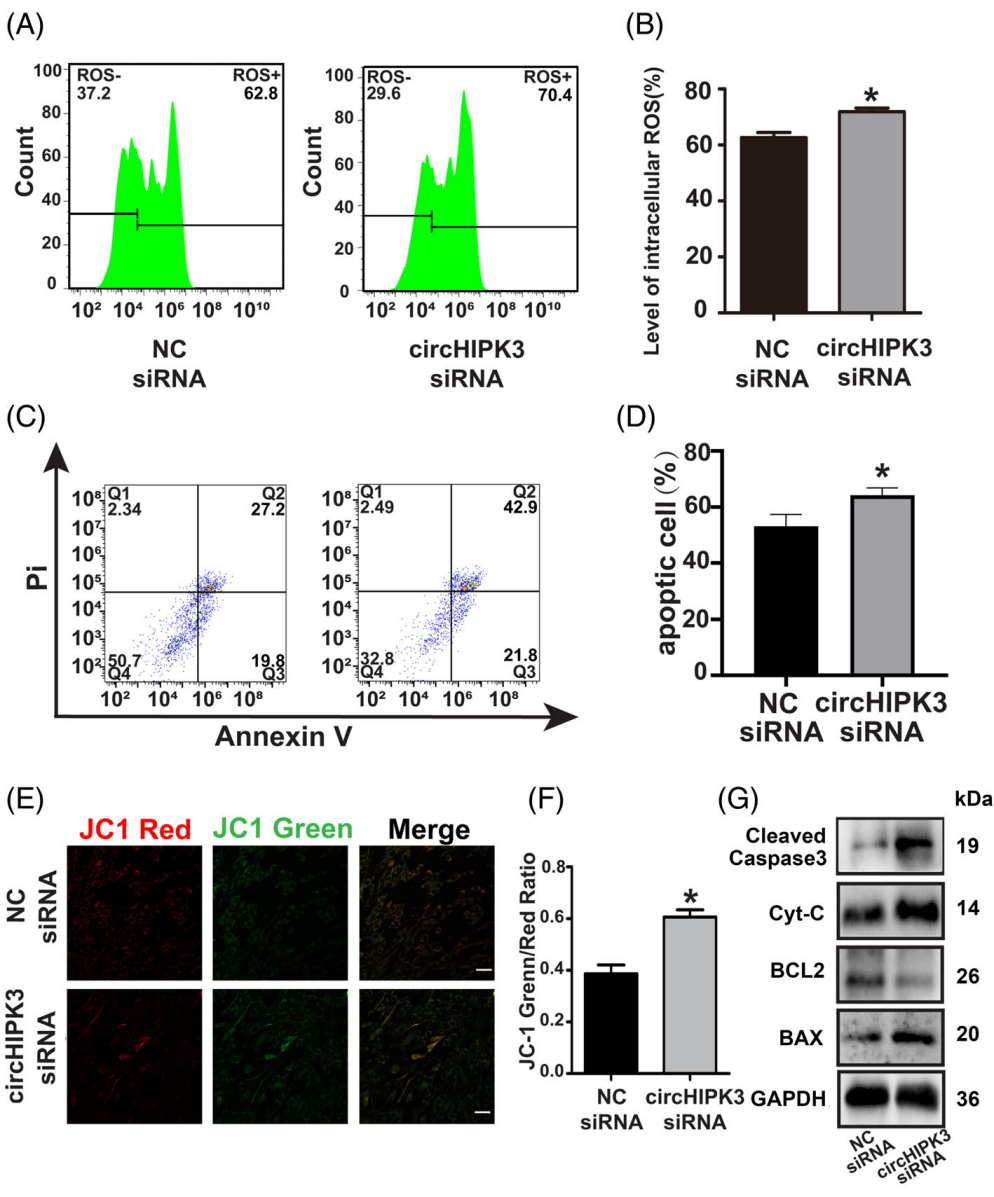
Downregulated circHIPK3 promoted intracellular ROS level, apoptosis and MOMP in HC‐a chondrocytes. (A, B) Flow cytometry showed intracellular ROS levels in HC‐a chondrocytes transfected with circHIPK3 siRNA (*n* = 3). (C, D) The effect of circHIPK3 on apoptosis was analyzed by flow cytometry (*n* = 3). (E) JC‐1 staining showed changes in mitochondria membrane potential. Green and red indicate JC‐1 monomers and JC‐1 aggregates, respectively. Bar =50 μm. (F) Quantification of JC‐1 green/red fluorescence ratio(*n* = 4). (G) Western blot analysis showed Cleaved‐Caspase 3, Cyt‐c, BCL‐2, BAX and GAPDH expressions in HC‐a chondrocytes transfected with circHIPK3 siRNA for 48 h. *p < 0.05

Since mitochondrial dysfunction is associated with ROS overproduction which subsequently activates the mitochondrial apoptotic pathways, so we first examined whether circHIPK3 regulated ROS production. The flow cytometry analysis showed a significantly increased ROS level when the circHIPK3 expression in HC‐a chondrocytes was inhibited (Figure 3A, B). Subsequently, we performed flow cytometry to analyze chondrocyte apoptosis by staining with Annexin V and PI. We observed a marked increase in apoptotic cell numbers in circHIPK3 siRNA transfected chondrocytes (Figure 3C, D). To confirm the increased apoptosis was caused by mitochondrial dysfunction, JC‐1 staining was applied to detect mitochondrial membrane potential. Mitochondrial depolarization was usually evaluated by the increase in the green/red fluorescence intensity ratio.^40^ Our results showed that compared with the NC group, knocking down circHIPK3 significantly increased the green/red ratio (Figure 3E, F). Furthermore, the expressions of the proteins associated with mitochondria apoptosis, such as BCL‐2, BAX, Cyt‐c, and Cleaved‐Caspase 3, were measured by western blot analysis. It was demonstrated that compared with the NC group, the expressions of BAX, Cyt‐c, and Cleaved‐Caspase 3 were promoted, while the expression of BCL‐2 was decreased when circHIPK3 was knocked down (Figure 3G). Western blot and flow cytometry analysis also revealed that the upregulation of circHIPK3 resulted in decreased apoptotic cells in IL‐1β treated HC‐a chondrocytes (Figure S2a–c). These results confirmed that circHIPK3 regulated mitochondria‐mediated apoptosis in HC‐a chondrocytes.

### 3.5 CircHIPK3‐related protein PON2 regulated mitochondria‐mediated apoptosis in HC‐a chondrocytes

**FIGURE 4:**
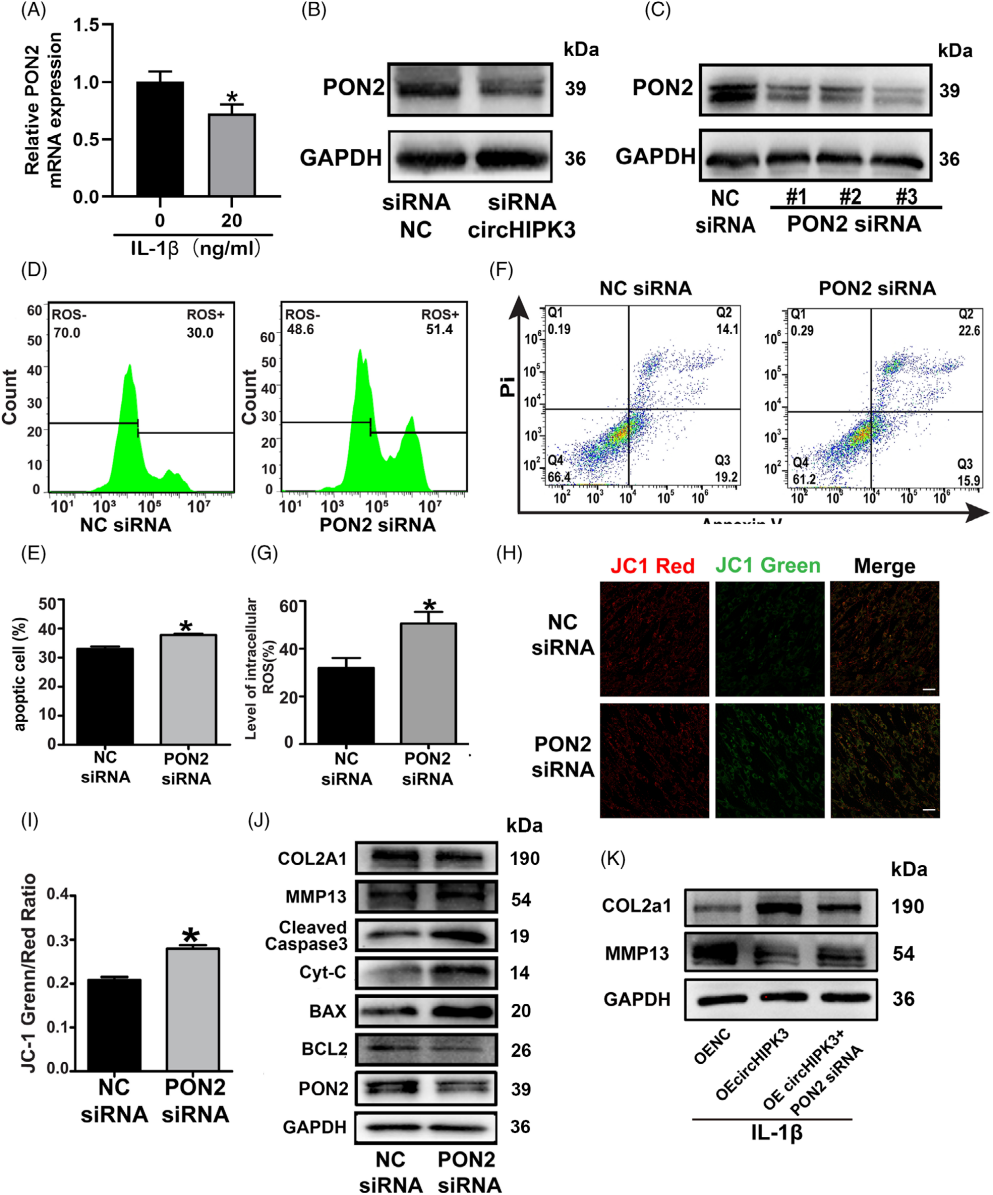
CircHIPK3 targeted gene PON2 regulated intracellular ROS level, apoptosis and MOMP in HC‐a chondrocytes. (A) The expression of PON2 in IL‐1β treated HC‐a chondrocytes was evaluated by RT‐qPCR. (B) Western blot analysis showed PON2 protein expression in HC‐a chondrocytes transfected with circHIPK3 siRNA for 48 h. (C) Western blot analysis showed PON2 protein expression in HC‐a chondrocytes transfected with PON2 siRNA #1,#2 and #3. (D, E) Flow cytometry showed intracellular ROS level in HC‐a chondrocytes transfected PON2 siRNA (*n* = 3). (F, G) The effect of knockdown of PON2 on apoptosis was analyzed by flow cytometry (*n* = 3). (H) JC‐1 staining showed changes in mitochondria membrane potential. Green and red indicate JC‐1 monomers and JC‐1 aggregates, respectively. Bar =50 μm. (I) Quantification of JC‐1 green/red fluorescence ratio(*n* = 4). (J) Western blot analysis showed Col2A1, MMP13, Cleaved‐Caspase3, Cyt‐c, BCL‐2, BAX and GAPDH expressions in HC‐a chondrocytes transfected PON2 siRNA for 48 h. (K) The expressions of Col2A1 and MMP13 were evaluated in HC‐a chondrocytes transfected with circHIPK3 siRNA and miR‐30a‐3p inhibitor for 48 h by Western blot. *p < 0.05

To further clarify the mechanism of circHIPK3 in regulating chondrocyte apoptosis, we focused on the altered circHIPK3‐related proteins obtained from proteomics studies. Among these proteins, PON2, an antioxidant enzyme, plays an important role in protecting against mitochondria‐mediated apoptosis.^41^ First, we detected the expression of PON2 in IL‐1β treated chondrocytes. Results of RT‐qPCR showed a significant decrease of PON2 in IL‐1β treated chondrocytes (Figure 4A). Then, we verified whether the expression of PON2 was regulated by circHIPK3. Western blot and RT‐qPCR analysis showed that expression of PON2 was decreased when circHIPK3 was knocked down (Figure 4B). Subsequently, we investigated the function of PON2 by knocking it down with siRNA in HC‐a chondrocytes. HC‐a chondrocytes were transfected with three different siRNAs, and it was found that siRNA #3 had the highest knockdown efficiency (termed as PON2 siRNA thereafter) (Figure 4C). Next, we analyzed the function of PON2 on chondrocyte apoptosis. Flow cytometry indicated that downregulation of PON2 by siRNA led to an increased ROS level in HC‐a chondrocytes (Figure 4D, E). Moreover, Annexin V and PI staining displayed increased apoptosis in HC‐a chondrocytes after PON2 was knocked down (Figure 4F, G). JC‐1 staining demonstrated a significant decrease in mitochondrial membrane potential after transfection of PON2 siRNA in HC‐a chondrocytes (Figure 4H, I). Results of western blot analysis revealed that when PON2 was knocked down, expressions of BAX, Cyt‐c, and Cleaved‐Caspase 3 were increased, while the expression of BCL‐2 was decreased (Figure 4J). Besides, decreased expression of COL2A1 and increased expression of MMP13 were also observed (Figure 4J). To further verify the regulatory relationship between circHIPK3 and PON2, we transfected circHIPK3 overexpression plasmids and PON2 siRNA in IL‐1β stimulated chondrocytes. The results showed the protective effect of circHIPK3 on the degradation of ECM (Figure 4K), intracellular ROS level (Figure S3a, b), apoptosis (Figure S3c–e), and mitochondrial membrane potential (Figure S3f, g) were abolished by knocking down PON2. All these results indicated that PON2 regulated mitochondria‐mediated apoptosis in HC‐a chondrocytes and was downstream of circHIPK3.

### 3.6 CircHIPK3 regulated PON2 expression by sponging miR‐30a‐3p in HC‐a chondrocytes

**FIGURE 5:**
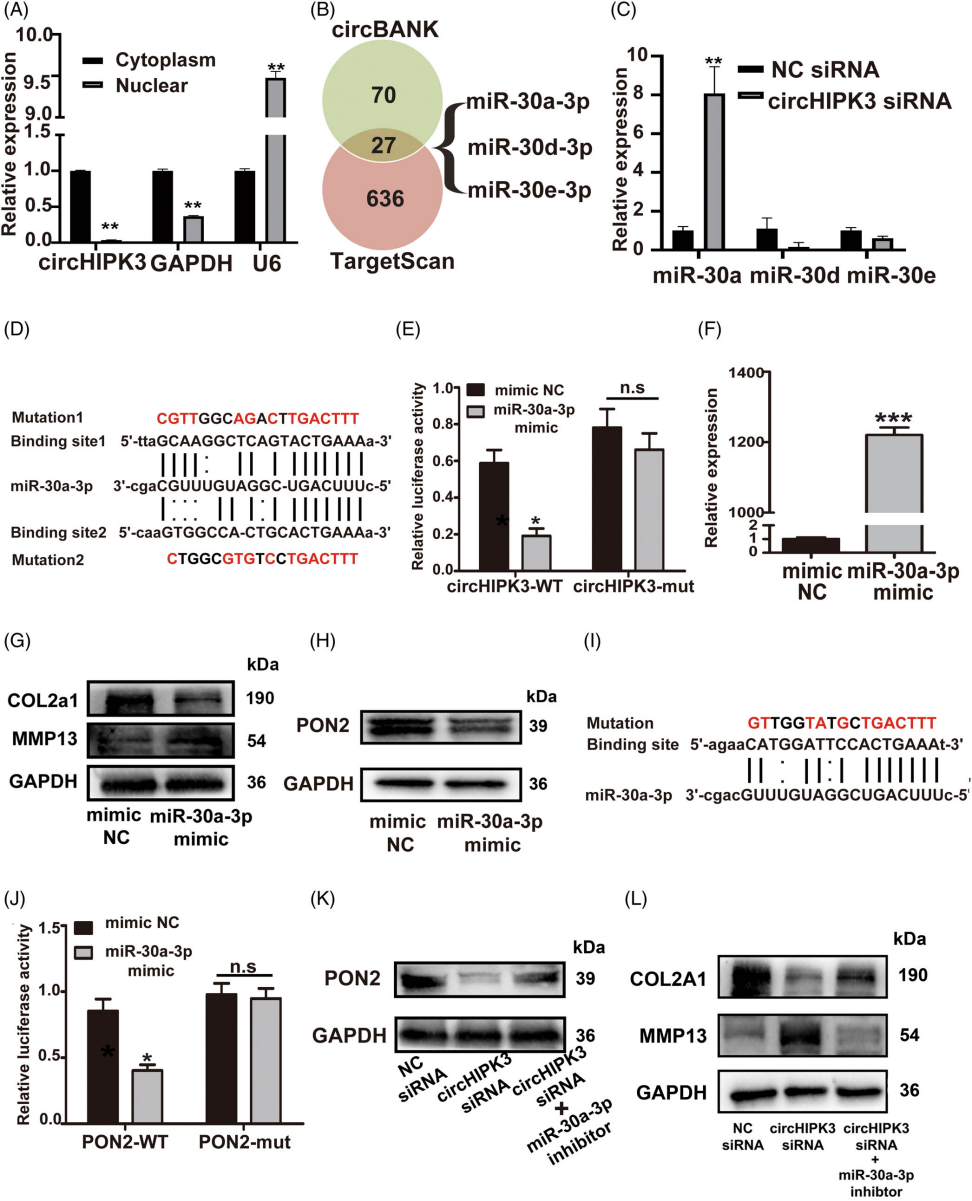
CircHIPK3 interacted with miR‐30a‐3p to regulate PON2 expression in HC‐a chondrocytes. (A) RT‐qPCR analysis showed circHIPK3, GAPDH mRNA and U6 expressions in cytoplasm and nuclear in HC‐a. (B) MiRNAs targeted circHIPK3 and PON2 were predicted by using of circBank, and TargetScan. (C) RT‐qPCR analysis showed miR‐30a‐3p, miR‐30d‐3p and miR‐30e‐3p expressions in HC‐a chondrocytes transfected with circHIPK3 siRNA. (D) The potential binding site sequence of miR‐30a‐3p on circHIPK3. The mutant sequences were highlighted in red. (E) Interaction between circHIPK3 and miR‐30a‐3p was verified by dual‐luciferase reporter assay. (F) RT‐qPCR analysis showed miR‐30a‐3p expression in HC‐a chondrocytes transfected miR‐30a‐3p mimic. (G), The expressions of Col2A1 and MMP13 were evaluated in HC‐a chondrocytes transfected with miR‐30a‐3p mimic. (H) Western blot analysis showed PON2 expression in HC‐a chondrocytes transfected with miR‐30a‐3p mimic. (I) The potential binding site sequence of miR‐30a‐3p on PON2. The mutant sequences were highlighted in red. (J) Interaction between PON2 and miR‐30a‐3p was verified by dual luciferase assay. (K, L) HC‐a chondrocytes were transfected with circHIPK3 siRNA and miR‐30a‐3p inhibitor for 48 h and western blot was conducted to detect the expressions of PON2, COL2A1 and MMP13. *p < 0.05, **p < 0.01, ***p < 0.001

Previous studies have reported the functional role of circHIPK3 as a miRNA sponge.^42^ To verify whether circHIPK3 was bound to miRNA in chondrocytes, we first investigated the distribution of circHIPK3, finding that circHIPK3 was mainly located in the cytoplasm of chondrocytes (Figure 5A). Then we conducted the bioinformatic analysis using circBank (http://www.circbank.cn/) and TargetScan (http://www.targetscan.org/) databases. CircHIPK3‐related miRNAs were sought by circBank, and the miRNAs regulating PON2 expression were predicted by TargetScan. A total of 27 miRNAs were predicted to be the target of circHIPK3. Among those miRNAs, we focused on miR‐30a‐3p, miR‐30d‐3p, and miR‐30e‐3p, which were the members of the miR‐30 family (Figure 5B, Table S4). MiR‐30a family has been reported to be involved in the development of bone tissue and OA pathogenesis.^43^ Further, we detected expressions of miR‐30a‐3p, miR‐30d‐3p, and miR‐30e‐3p in HC‐a chondrocytes and RT‐qPCR revealed that miR‐30a‐3p was markedly increased after knocking down circHIPK3. (Figure 5C). Moreover, we found that circHIPK3 has two potential binding sites of miR‐30a‐3p (Figure 5D). To confirm the interaction between circHIPK3 and miR‐30a‐3p, the dual‐luciferase reporter assay was performed. CircHIPK3 sequences containing two miR‐30a‐3p binding sites or the mutated sequences were inserted into psiCHECK2 vectors. Recombinant vectors with wild‐type sequence (circHIPK3‐WT) were co‐transfected into 293T cells with miRNA‐30a mimic or mimic NC. Meanwhile, as the control groups, the psiCHECK2 vectors containing mutated sequences (circHIPK3‐mut) were also co‐transfected into 293T cells with miRNA‐30a mimic or mimic NC. The activity of luciferase in the group transfected with circHIPK3‐WT and miR‐30a‐3p mimic was significantly decreased compared to the group transfected with circHIPK3‐WT and mimic NC. However, there was no significant difference in the control groups (Figure 5E). To reveal the function of miR‐30a‐3p in chondrocytes, HC‐a chondrocytes were transfected with miR‐30a‐3p mimic (Figure 5F). Degradation of ECM (Figure 5G), increased ROS levels (Figure S4a, b), promoted apoptosis (Figure S4c–e) and mitochondrial depolarization (Figure S4f, g) were observed in HC‐a chondrocytes when miR‐30a‐3p was overexpressed (Figure S4). These results indicated that circHIPK3 might directly bind to miR‐30a‐30 in HC‐a chondrocytes.

Furthermore, we evaluated the effect of miR‐30a‐3p on PON2 expression. Western blot showed that PON2 expression was decreased when transfecting miR‐30a‐3p mimic in HC‐a chondrocytes (Figure 5H). Interestingly, the binding sites of miR‐30a‐3p were found in PON2 (Figure 5I). Then we explored whether miR‐30a‐3p is bound to PON2 by dual‐luciferase reporter assay. The activity of luciferase of the group transfected with vectors containing wild type PON2 (PON2‐WT) sequence and miR‐30a‐3p mimic was significantly decreased compared to the group transfected with PON2‐WT and mimic NC (Figure 5J). However, luciferase activity showed no significant difference in the control groups co‐transfected with vectors containing mutated PON2 sequence (PON2‐mut) and miR‐30a‐3p mimic or mimic NC (Figure 5J). Finally, HC‐a chondrocytes were co‐transfected with circHIPK3 siRNA and/or miR‐30a‐3p inhibitors. Western blot analysis demonstrated that silencing miR‐30a‐3p restored the effect of circHIPK3 knockdown on PON2 expression (Figure 5K), as well as the expressions of COL2A1, MMP13 (Figure 5L), Cleaved‐Caspase3, Cyt‐c, BCL‐2, and Bax (Figure S5a). These results demonstrated that circHIPK3 directly interacted with miR‐30a‐3p to regulate PON2 in HC‐a chondrocytes.

### 3.7 Overexpression of circHIPK3 decreased cartilage degradation in mouse OA model

**FIGURE 6:**
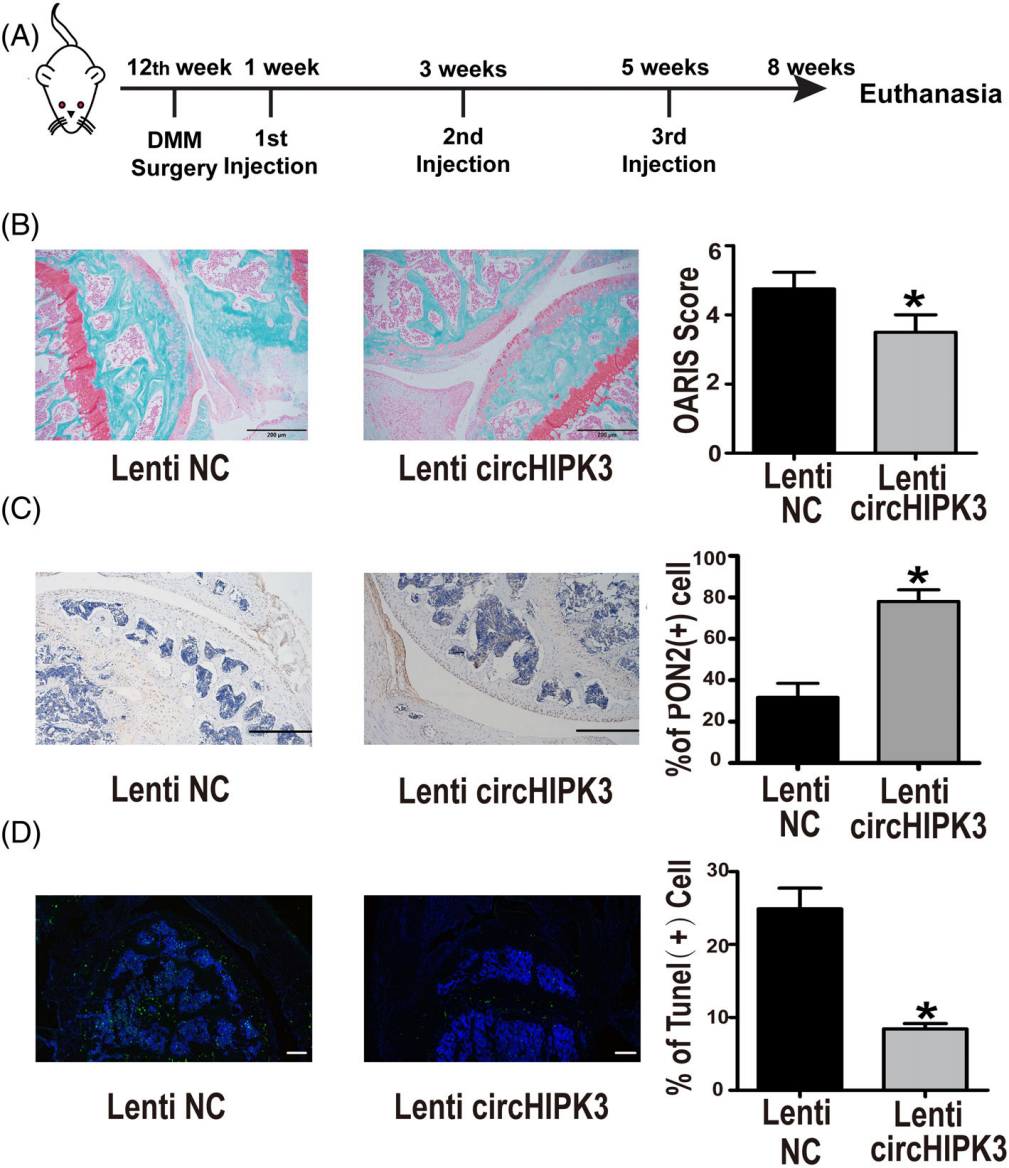
Overexpression of circHIPK3 alleviated the degradation of cartilage in DMM mice. (A) Schematic of mouse DMM model construction and lentivirus intra‐articular injection. (B) Cartilage destruction was evaluated by safranin O‐fast green staining and quantified by OARSI grade. (C) PON2 immunostaining was performed in cartilage and PON2 positive cells were quantified. (D) Tunel assay was performed and apoptotic cells were counted. DMM mice were injected with Lenti‐NC (*n* = 4) as a control or Lenti‐circHIPK3 (*n* = 4). *p < 0.05, Bar = 200 μm

To evaluate the effect of circHIPK3 on cartilage degradation in vivo, the mouse OA model was constructed by DMM surgery. CircHIPK3 (has_circ_000284), located at chr11:33307958 to 33309057 in the human genome, was highly conserved with mmu_circ_0001052, which was derived from chr2:104310905 to 104312004 of mouse genome.^20^ Lentivirus was injected intra‐articularly at weeks 1, 3, and 5 after DMM surgery to overexpress mmu_circ_0001052 for imitating the function of has_circHIPK3 in vivo (Figure 6A). Sections were safranin O‐fast green stained, and cartilage degradation was assessed by OARSI grade. The results showed that cartilage destruction was suppressed when circHIPK3 was upregulated (Figure 6B). Elevated expression of PON2 was also observed in the Lenti circHIPK3 groups compared with control groups (Figure 6C). Besides, we performed TUNEL staining to examine the effect of circHIPK3 on apoptosis in vivo. The results revealed that overexpressing circHIPK3 decreased the number of TUNEL positive cells in the cartilage (Figure 6D). Taken together, these results demonstrated that overexpression of circHIPK3 could inhibit apoptosis and cartilage destruction in the DMM OA mouse model.

## 4 DISCUSSION

OA is a common joint disease featured by cartilage degradation.^1^ Chondrocytes are the unique cellular component of cartilage and regulate the balance between catabolic and anabolic activities to keep cartilage homeostasis.^44^, ^45^ Currently, growing studies have indicated that circRNAs are involved in OA progress by regulating catabolic and anabolic activities of chondrocytes.^46^, ^47^, ^48^ In this study, we found that circHIPK3 was downregulated in OA chondrocytes and might play an essential role in chondrocyte apoptosis by regulating mitochondrial depolarization.

Apoptosis of chondrocytes, closely related to mitochondrial dysfunction, results in disruption of the balance between catabolic and anabolic processes in cartilage.^8^, ^12^ During apoptosis, the increment of ROS causes MOMP, which induces the release of Cyt‐c.^49^ Subsequently, the released Cyt‐c activates Caspase 3, the apoptosis executor enzyme.^50^ In addition, apoptotic stimuli also change the balance between anti‐apoptotic and pro‐apoptotic proteins, which also contributes to mitochondria‐mediated apoptosis.^51^ Previous studies have revealed that circHIPK3 regulated apoptosis in multiple diseases.^52^, ^53^, ^54^ In renal carcinoma cells, circHIPK3 downregulated expressions of Cleaved‐Caspase3 and Bax and upregulated expression of Bcl‐2.^53^ In this study, we performed proteomics studies and Gene Ontology analysis. We found oxidative activity‐related terms were highly ranked and some mitochondria‐related proteins were detected. Furthermore, downregulated circHIPK3 in chondrocytes increased intracellular ROS, promoted MOMP, and induced cell apoptosis. Above all, these results suggested that circHIPK3 regulated chondrocyte apoptosis by mitochondria‐mediated pathway, which is consistent with previous research.

To further explore the mechanism of circHIPK3 regulating chondrocyte apoptosis by mitochondrial pathway, we identified PON2 as a potential target of circHIPK3. Previous studies have indicated that PON2 functioned as a negative regulator of apoptosis in various cells.^30^, ^34^ Asokan et al. have reported that PON2 protein was localized in mitochondria inner membrane and reduced oxidative stress by regulating respiratory chain complex III activities.^30^ Moreover, PON2 prevented mitochondrial ROS synthesis and apoptosis in human endothelial cells.^36^ Herein, we found that circHIPK3 regulated PON2 expression in HC‐a chondrocytes, and PON2 expression was downregulated in IL‐1β treated chondrocytes. Furthermore, silencing PON2 expression increased intracellular ROS levels and apoptosis in HC‐a chondrocytes. These findings suggested that PON2 might be a potential target of circHIPK3 in chondrocytes to regulate apoptosis. Besides PON2, we also found other proteins related to mitochondria in proteomics studies, such as Glutathione S‐transferase kappa 1 (GSTK1), frataxin (FXN), FAD‐dependent oxidoreductase domain‐containing protein 1 (FOXRED1), Apoptosis‐inducing factor 3 (AIFM3), and Peroxiredoxin‐like 2A (PRXL2A). These proteins implied that other mechanisms might also be involved in circHIPK3 regulated chondrocyte apoptosis. Further studies are still required to fully investigate the functional role of circHIPK3 in OA.

Recently, increasing numbers of studies have shown that circRNAs could act as miRNA sponges for binding miRNAs to target mRNAs.^55^, ^56^ Previous studies have shown that miR‐30a‐3p might be the target of circHIPK3 in mice^20^ and chicken.^57^ In this study, we found that both circHIPK3 and PON2 had miR‐30a‐3p binding sites, which was verified by luciferase assay and rescue experiment. In addition, overexpressing miR‐30a‐3p promoted intracellular ROS levels and apoptosis in HC‐a chondrocytes. Above all, these results implied that circHIIPK3 might serve as a sponge of miR‐30a‐3p to regulate PON2 expression and apoptosis in chondrocytes.

In summary, our study portrayed a functional role of circHIPK3 in chondrocytes and OA progress. We showed that circHIPK3 regulated chondrocytes apoptosis through mitochondrial pathway and circHIPK3/miR‐30a‐3p/PON2 axis might be a potential strategy for OA therapeutics.

## AUTHOR CONTRIBUTIONS

X.X. conceived the study and participated in the study design, performance, and manuscript writing; J.S., H.L., B.W., and J.N performed experiments and analyzed the data; D.W., B.W. and J.Z. provided techniques and discussion; X.X and J.S wrote the manuscript. All authors read and approved the final manuscript.

## FUNDING INFORMATION

This study was supported by grants from the National Natural Science Foundation of China (NO. 81772384, 81572174, and 81902242), China Postdoctoral Science Foundation (NO. 2019M663268), Guangdong Medical Research Foundation (NO.A2020283), Medical Science and Technology Planning Project of Zhuhai, China (NO.ZH2201200003HJL) and Science and Technology Project of Jiangxi Provincial Education Department (NGJJ210176). The funders had no roles in the study design, data collection, data analysis, manuscript preparation, or publishing decision.

## CONFLICT OF INTEREST

The authors declare no competing interests.

## Supporting information


Figure S1

Figure S2

Figure S3

Figure S4

Figure S5

Table S1

Table S2

Table S3

Table S4

## Data Availability

The data that support the findings of this study are available from the corresponding author upon reasonable request.
