# Supplementary figures and images for: CircHIPK3 prevents chondrocyte apoptosis and cartilage degradation by sponging miR‐30a‐3p and promoting PON2
Source: Cell Prolif. 2022 Jun 18;55(9):e13285. doi: 10.1111/cpr.13285 (PMC9436899; doi:10.1111/cpr.13285)

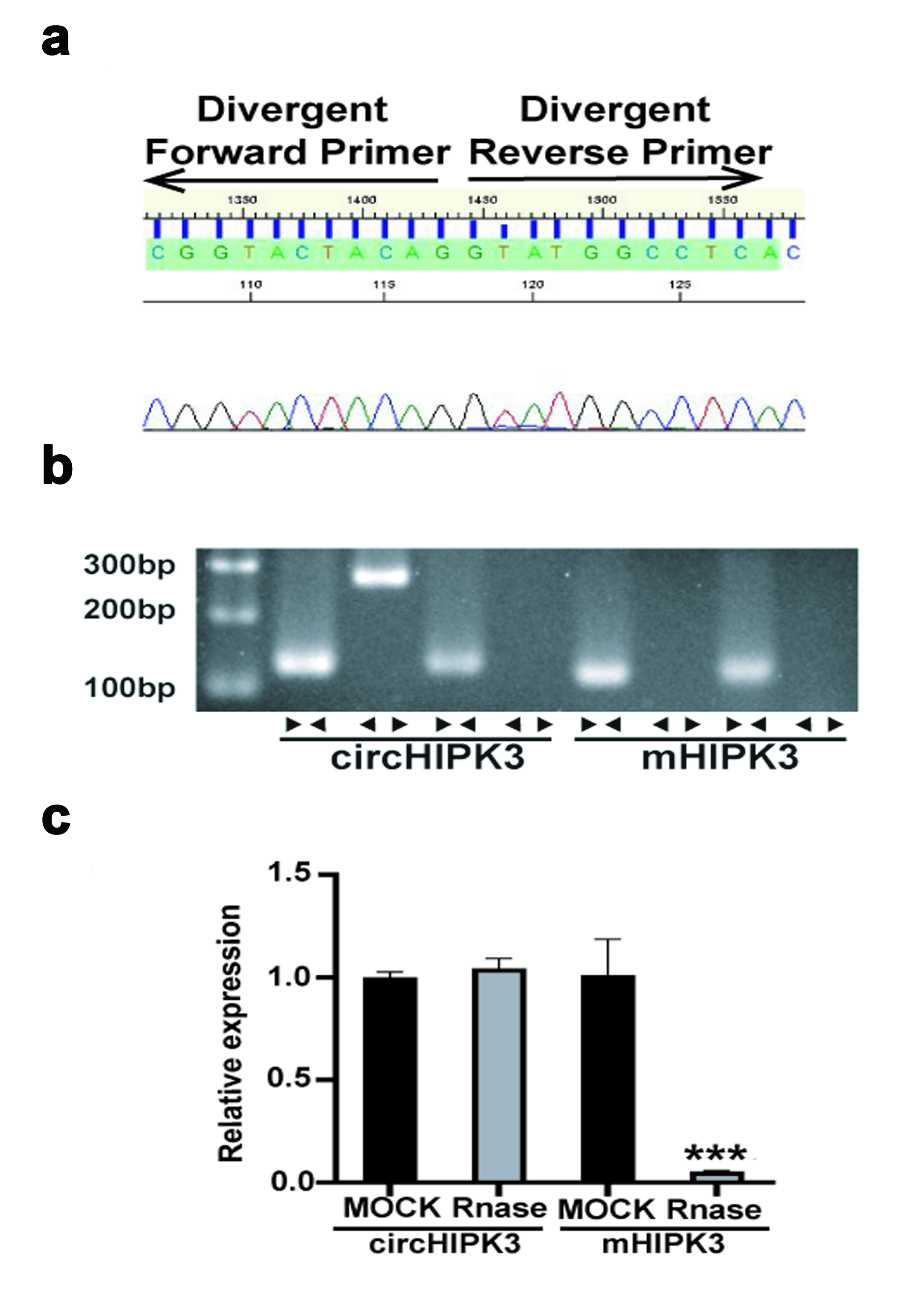

Supplement: Supplementary file 1 — Figure S1 [file CPR-55-e13285-s005.tif]

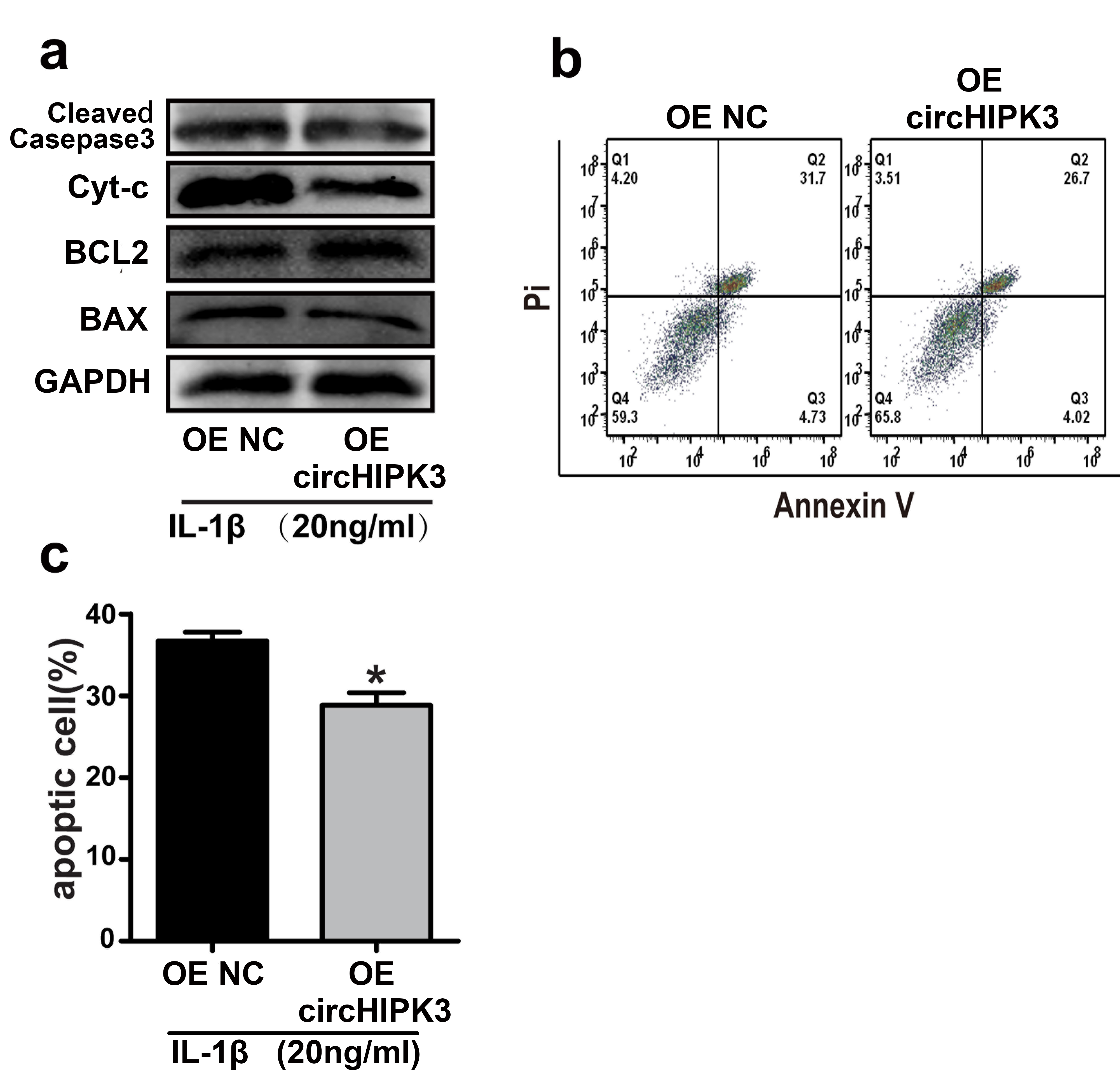

Supplement: Supplementary file 2 — Figure S2 [file CPR-55-e13285-s007.tif]

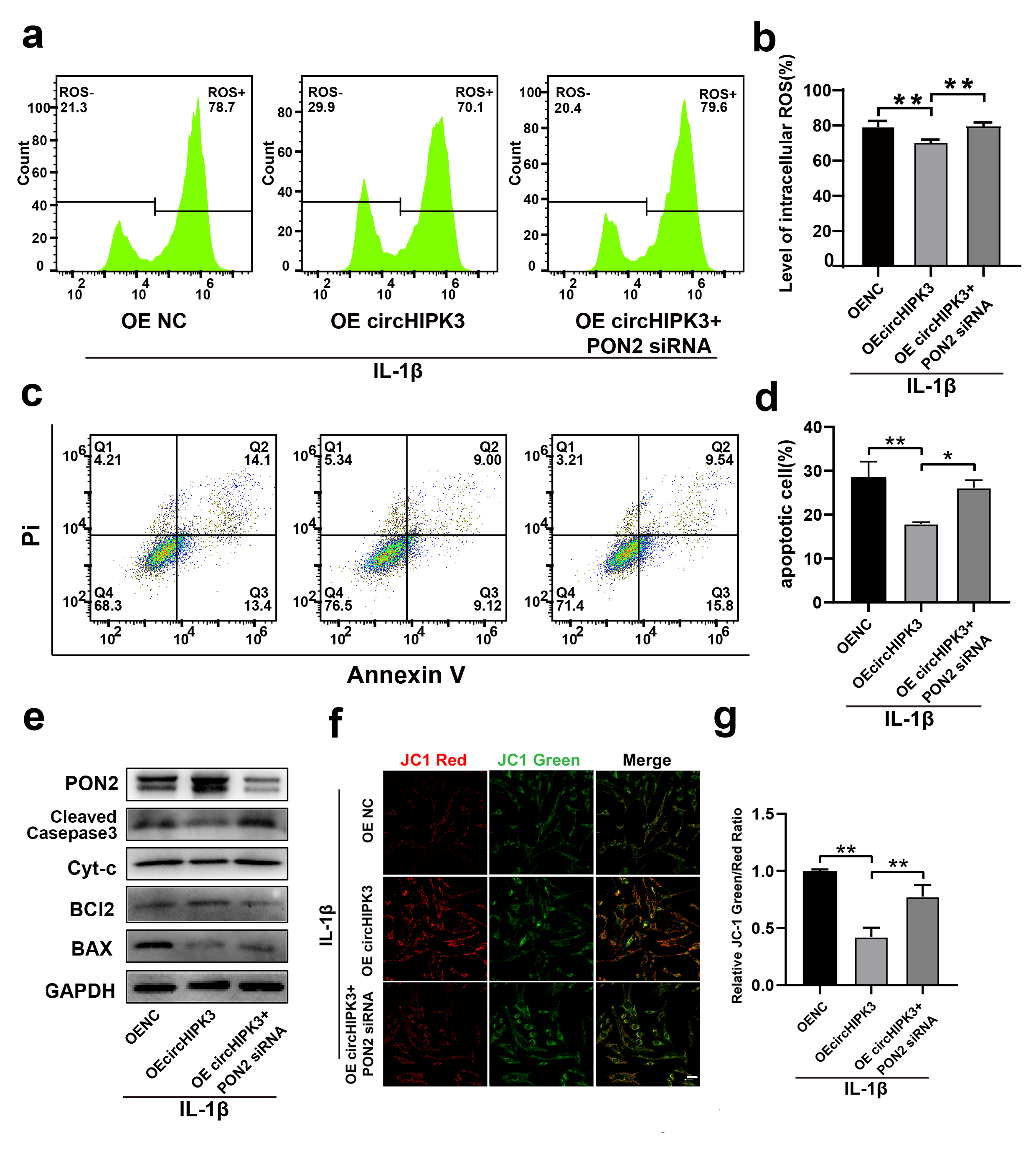

Supplement: Supplementary file 3 — Figure S3 [file CPR-55-e13285-s009.tif]

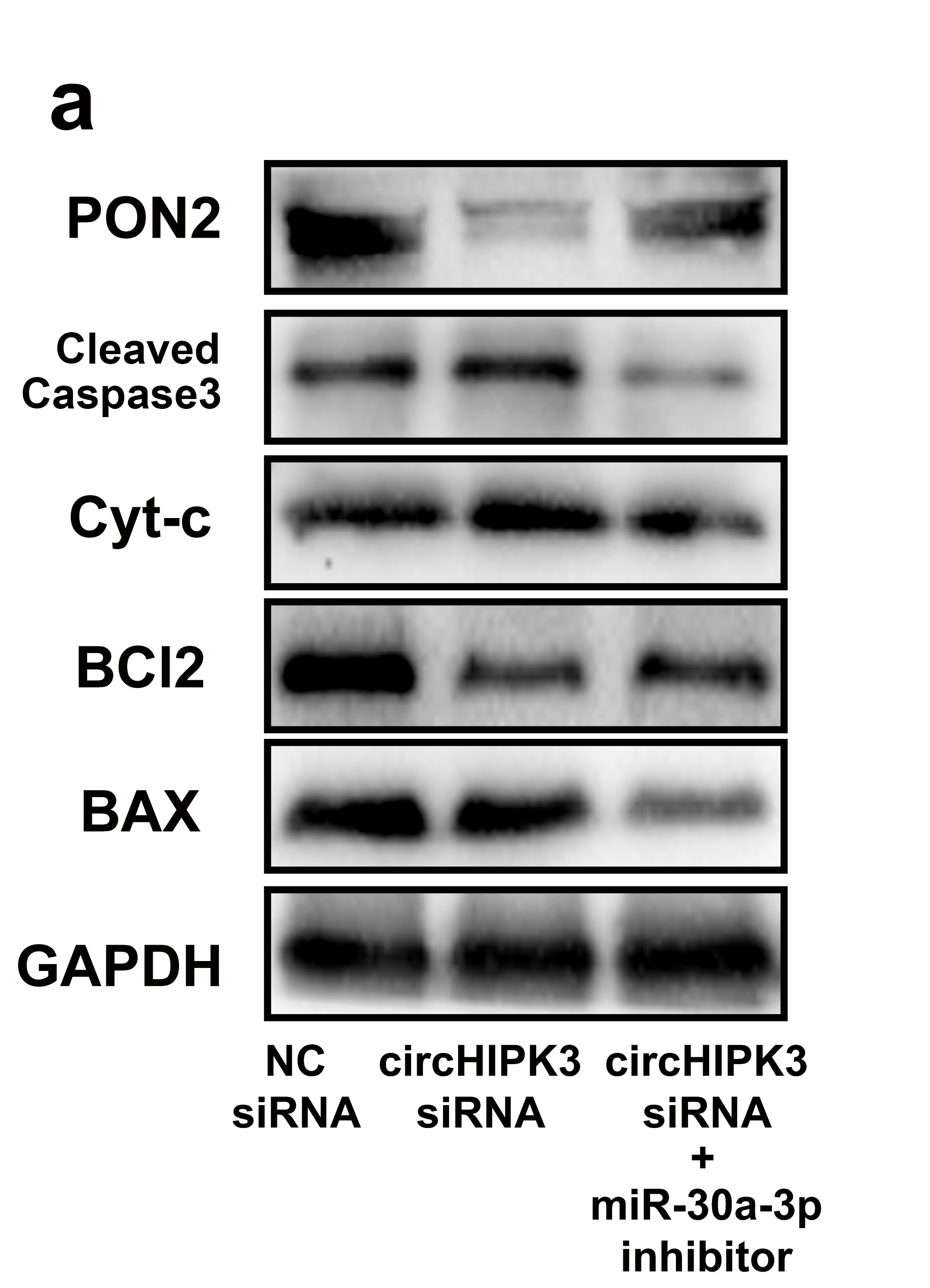

Supplement: Supplementary file 5 — Figure S5 [file CPR-55-e13285-s006.tif]
