# Supplementary material for: CircHIPK3 prevents chondrocyte apoptosis and cartilage degradation by sponging miR‐30a‐3p and promoting PON2
Source: Cell Prolif. 2022 Jun 18;55(9):e13285. doi: 10.1111/cpr.13285 (PMC9436899; doi:10.1111/cpr.13285)
Supplement: Supplementary file 6 — Table S1 [file CPR-55-e13285-s004.docx]

Supplementary Table S1 Primers and sequences used in this study

| **Primers** | | | |
| --- | --- | --- | --- |
| hsa_circHIPK3 | Forward | | TATGTTGGTGGATCCTGTTCGGCA |
|  | Reverse | | TGGTGGGTAGACCAAGACTTGTGA |
| mHIPK3 | Forward | | TGGAGACTGGGGGAAGATGA |
|  | Reverse | | CACACTAACTGGCTGAGGGG |
| COL2A1 | Forward | | CCCATGTGGAAGGCAGATG |
|  | Reverse | | TTCTGAGAGGCACAGGTGACA |
| MMP13 | Forward | | CACTTTATGCTTCCTGATGACG |
|  | Reverse | | TCCTCGGAGACTGGTAATGG |
| PON2 | Forward | | TCTGGCACTCAGAAATCGACT |
|  | Reverse | | TTAATTCCCGTGCCCTTGGTT |
| GADPH | Forward | | AGCCACATCGCTCAGACAC |
|  | Reverse | | GCCCAATACGACCAAATCC |
| hsa_miR-30a-3p | Forward | | CAGTGCTGCTTTCAGTCGGAT |
|  | Reverse | | TATGGTTGTTCACGACTCCTTCAC |
| hsa_miR-30d-3p | Forward | | CCGTCFCTTTCAGTCAGATG |
|  | Reverse | | AGAGCAGGGTCCGAGGAT |
| hsa_miR-30e-3p | Forward | | TGCTGTTTCAGTCGGATG |
|  | Reverse | | CACTTCCTCTGCACTTGTTGGTAT |
| U6 | Forward | | CTCGCTTCGGCAGCACA |
|  | Reverse | | GCGAGCACAGAATTAATACGAC |
| Divergent GAPDH primer | Forward | | TCCTCACAGTTGCCATGTAGACCC |
|  | Reverse | | TGCGGGCTCAATTTATAGAAACCGGG |
| Convergent hsa_circHIPK3 primer | Forward | | TCACAAGTCTTGGTCTACCCA |
|  | Reverse | | CACATAGGTCCGTGGATAGTTTC |
| mmu_circ_0001052 | Forward | | GGATCGGCCAGTCATGTATC |
|  | Reverse | | ACCGCTTGGCTCTACTTTGA |
| mmu GAPDH | Forward | | GTCAAGGCTGAGAACGGGAA |
|  | Reverse | | AAATGAGCCCCAGCCTTCTC |
| **SiRNAs** | | | |
| NC siRNA | Forward | | ACGUGACACGUUCGGAGAAdTdT |
|  | Reverse | | ACGUGACACGUUCGGAGAAdTdT |
| hsa_circHIPK3 siRNA#1 | Forward | | UCGGUACUACAGGUAUGGCdTdT |
|  | Reverse | | GCCAUACCUGUAGUACCGAdTdT |
| hsa_circHIPK3 siRNA#2 | Forward | | GUACUACAGGUAUGGCCUCdTdT |
|  | Reverse | | GAGGCCAUACCUGUAGUACdTdT |
| hsa_circHIPK3 siRNA#3 | Forward | | CUACAGGUAUGGCCUCACAdTdT |
|  | Reverse | | UGUGAGGCCAUACCUGUAGdTdT |
| PON2 siRNA #1 | Forward | | GCACUCAGAAAUCGACUUATT |
|  | Reverse | | UAAGUCGAUUUCUGAGUGCTT |
| PON2 siRNA #2 | Forward | | CCACCUGAUUAAAGGAAUUTT |
|  | Reverse | | AAUUCCUUUAAUCAGGUGGTT |
| PON2 siRNA #3 | Forward | | GCUUCUUCCAAGUGUGAAUTT |
|  | Reverse | | AUUCACACUUGGAAGAAGCTT |
| Other sequences | | | |
| miRNA mimic NC | | UUCUCCGAACGUGUCACGUTT | |
| miR-30a-3p mimic | | CUUUCAGUCGGAUGUUUGCAGC | |
| miR-30a-3p inhibitort | | GCUGUAAACAUCCGACUGAAAG | |
